# Delayed onset of neonatal compartment syndrome associated with compound fetal presentation

**DOI:** 10.1186/s12887-023-04505-0

**Published:** 2024-04-01

**Authors:** Nicholas Manini, Hayato Unno

**Affiliations:** 1 Alfred I. duPont Hospital for Children, 1600 Rockland Road, Wilmington, DE, 19803 USA; 2 Thomas Jefferson University Hospital, 111 S 11th St, Philadelphia, PA 19107 USA

**Keywords:** Neonatal compartment syndrome, Compound delivery, Fasciotomy

## Abstract

Neonatal compartment syndrome, although rare, has a classic presentation with sentinel skin findings and development of swelling, erythema, and tenderness of the affected extremity. Neonatal compartment syndrome requires prompt surgical intervention to preserve the affected limb and ensure its normal growth and development. Our patient was born at term via vaginal delivery complicated by a compound presentation involving the left upper extremity. No physical exam abnormalities were noted at birth, but she developed signs of neonatal compartment syndrome by 15 h of life. She was surgically treated at 22 h of life and recovered well. At one year of age, she has normal growth and function of the affected extremity. Our case adds to the growing literature associating neonatal compartment syndrome with a compound fetal presentation.

## Background

A compound presentation occurs when a fetal extremity presents with the part of the fetus closest to the birth canal. Most commonly, an arm or hand presents adjacently to the head while the fetus is in vertex position. Complications of compound delivery include umbilical cord prolapse, shoulder dystocia, and injury to the affected limb [1]. Acute compartment syndrome, a potential sequalae of limb injuries, occurs when pressure builds within a closed osteofascial compartment due to decreased compartmental space or increased fluid volume within the compartmental space [2, 3]. Symptoms include pain, pulselessness, paresthesia, paralysis, and pallor, especially in older patients. However, neonatal compartment syndrome presents differently. Typically, skin changes are present, including erythema, edema, discoloration, and even gangrene [2, 4–6]. The forearm is the most frequently affected extremity in neonates [4]. Neonatal compartment syndrome is quite rare, with fewer than 100 cases reported in the literature [5]. There is no clear difference in its incidence in developed countries versus developing countries, but it is likely underreported in the latter. Etiologies can be extrinsic or intrinsic. Extrinsic etiologies are usually those that cause injury to the extremity, such as oligohydramnios, umbilical loops, amniotic bands, or birth trauma. Intrinsic causes are often associated with a hypercoagulable state, such as arterial thromboembolism [4, 5]. Neonates do not have established pressure gradients, so measurement of compartment pressures in this population remains controversial. Emphasis remains on clinical diagnosis [6, 7]. If not treated appropriately, neonatal compartment syndrome can lead to fibrosis of muscles and nerves, Volkmann contracture, abnormal limb development, and the need for amputation [5]. In a case series of twenty-four patients conducted by Ragland et al., only one patient showed full recovery [4]. Our patient is a full-term female who was found at 15 h of life to have compartment syndrome of the left forearm, secondary to injury from a compound presentation during labor and vaginal delivery.

## Case presentation

**Fig. 1:**
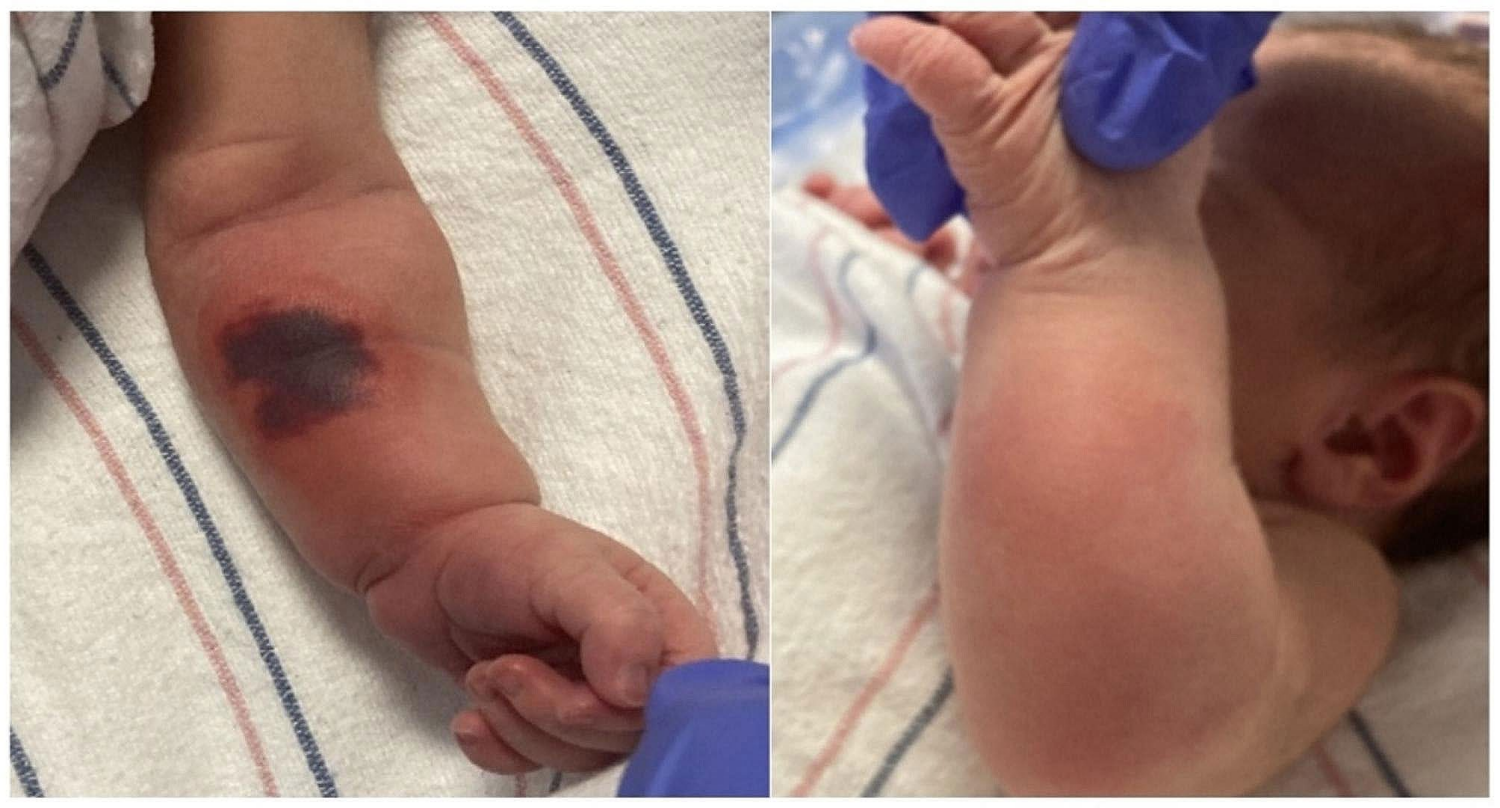
Volar and dorsal aspects of left forearm at approximately 15 h of life

A 2.88-kilogram female infant born at 39 1/7 weeks gestation via vaginal delivery was found at 15 h of life to have a non-blanching, violaceous patch on the volar aspect of her left mid forearm. Mild erythema and edema were present and extended to the dorsal aspect of the forearm (Fig. 1). She was born to a 31-year-old G1P0 mother with iatrogenic hypothyroidism secondary to Graves’ disease status post thyroid ablation two years prior. The mother also had a history of HSV-2 infection but did not have an outbreak during the pregnancy. Medications taken during the pregnancy included Levothyroxine, Valacyclovir, Ondansetron, Prochlorperazine, prenatal vitamins, Propranolol, and Senna. All routine prenatal labs and ultrasounds were normal. During an elective induction of labor, a fetal compound hand presentation was first noted by the obstetrics provider on vaginal exam seven hours prior to delivery. At that time, the cervix was 7.5 cm dilated, 80% effaced, and the fetal station was − 2. The compound hand was noted again on a repeat vaginal exam five hours prior to delivery. Reduction was not attempted because the cervix was not yet fully dilated. Ultimately, the baby was delivered vaginally in direct occiput anterior position with a fetal left hand compound presentation. The hand, shoulders, and body were delivered easily with maternal expulsive efforts. There was no nuchal cord or cord prolapse. APGAR scores were 8 and 9 at 1 and 5 min of life. On assessment by the pediatric team at 15 h of life, the infant was noted to have the aforementioned skin findings on her left forearm. Palpation and manipulation of the left upper extremity elicited tenderness and fussiness, but she was calm and consolable during other parts of the exam. There was weak grasp reflex of the left hand compared to the right, and her Moro reflex was asymmetric. No signs of Erb’s palsy were present. Capillary refill was less than 2 s in each digit of the left hand, and there was no temperature difference between extremities. Her vital signs, while she was calm, were temperature 36.8 degrees Celsius, heart rate 120 beats per minute, respiratory rate 40 breaths per min. Length was 50 centimeters (68th percentile based on WHO Girls 0–2 years). Head circumference was 33 centimeters (23rd percentile based on WHO Girls 0–2 years). The remainder of her physical exam was unremarkable.

X-ray films of the left upper extremity and the left clavicle showed no acute bony fracture or dislocations. Hand Surgery evaluated the patient at 18 h of life and made a clinical diagnosis of neonatal compartment syndrome. Ultrasound with doppler was not performed. She was emergently transferred to a pediatric hospital for fasciotomy. At the receiving hospital, capillary refill of the left hand was increased to 3 s. Ulnar and radial pulses in the left upper extremity were 1+, faint but detectable, and there was evolving skin discoloration of the left forearm. Cellulitis was considered, so blood cultures were obtained prior to the administration of peri-operative Cefazolin. Compartment pressures were not obtained. Decompressive fasciotomy was performed under general anesthesia at 22 h of life. A volar incision was made through the area of maximal compression, extending distally towards the wrist and proximally toward the medial aspect of the antecubital fossa. It was noted that upon incision of the muscle fascia and release of the volar forearm compartment, the muscles were yellow tan in appearance. Once the entire compartment was released, the muscle returned to normal perfused color. The skin was partially closed with the central third of the incision left open because the skin was of marginal viability. The wound was covered with Polysporin followed by Adaptic and dry gauze.

The infant had an uncomplicated post-operative course in the neonatal intensive care unit. Post-operative pain was controlled with Acetaminophen and Nalbuphine. She did not receive additional systemic antibiotics, and the blood culture remained without growth. Bilirubin levels were checked, and the infant did not require phototherapy. Due to the maternal history of Graves’ disease, Thyroid Stimulating Hormone (TSH) and Free Thyroxine (T4) were obtained on day of life two and were within age-appropriate limits. The patient was discharged on day of life four with Kling wrap dressing. The parents were able to perform wound care and dressing changes at home. Outpatient medications included acetaminophen as needed for pain and Cholecalciferol. Of note, cord blood Thyroid Stimulating Hormone Receptor Antibody testing ordered at the birth hospital resulted 5.76 IU/L (reference ≤ 2.00 IU/L). At the follow up visits with the primary care provider, clinical suspicion for thyrotoxicosis remained low as the patient did not exhibit signs or symptoms, and she was gaining weight appropriately. With the guidance of pediatric endocrinology, TSH and Free T4 levels were checked twice more (on days of life 13 and 24) with normal results. The state newborn screen was normal. The patient also had outpatient follow-up with hand surgery, occupational therapy, and Early Intervention. As the surgical wound healed, she wore an upper extremity orthotic brace for 8 h per day for one month to prevent contractures. Most recently, at her twelve months well visit, she had normal range of motion and function of her left upper extremity. Other than a visible surgical scar on the forearm, no extremity deformities were present.

## Discussion

Neonatal compartment syndrome is extremely rare, but several cases associated with compound presentations have been reported [6, 8, 9]. In all the previously reported cases, the infants had clear clinical manifestations of compartment syndrome immediately after birth [7, 9–12]. Our patient displayed no obvious signs or symptoms of compartment syndrome at birth. Her skin and neurovascular changes developed in the hours after and continued to acutely evolve, prompting urgent escalation of care. The patient’s mother received complete and appropriate prenatal surveillance, and the fetal compound presentation was first documented during labor, seven hours prior to delivery. There was no prolonged labor or arrest of descent. The presumed timing and duration of the injury may explain the unique presentation of our case, which likely represents the initial and early stages of neonatal limb compartment syndrome due to an extrinsic cause. No literature suggests any link between the mother’s medical conditions or medications with fetal malpresentation or compartment syndrome. With timely diagnosis and surgery, our patient had a favorable outcome with normal function and range of motion of the affected extremity at 12 months of age. Clinicians should consider a fetal compound presentation, at any point prior to delivery, a risk factor for neonatal compartment syndrome and should closely monitor for signs of it in the hours after birth, even if the initial exam at birth is normal. The presentation of neonatal compartment syndrome can vary in timing but is remarkably consistent with the appearance of a sentinel lesion and other skin changes such as discoloration, desquamation, blistering, or skin necrosis [5, 6]. The affected limb also becomes tender, tense, swollen and eventually progresses to motionlessness and pulselessness [6, 10]. The normal timeline for neonatal compartment syndrome has not been well documented due in part to its extreme rarity, but our case offers new insight. Prompt recognition and treatment with fasciotomy are critical in these patients as irreversible muscle and nerve damage occurs within 8 h of ischemia [10]. Swift intervention also prevents complications such as Volkmann contractures, bone development abnormalities, neuropathies, scar contractures, and the need for amputation [6, 7, 9]. Ragland et al. compiled a case series of 24 patients who suffered from neonatal compartment syndrome. Of the 24 patients, only one underwent fasciotomy within the first 24 h of life and was the only patient without subsequent complications [4]. Diagnosis remains a challenge due to its rarity and because neonatal compartment syndrome can mimic other diseases, such as cellulitis [10]. As such, delay in diagnosis is the most common cause for delayed surgical intervention [4]. After surgical intervention, our patient was closely followed by the surgical team, occupational therapy, and her primary care provider. Post-operative interventions, including stretching, bracing, scar massaging, and Early Intervention services also likely played a significant role in our patient’s successful recovery. Our case adds to the growing literature associating neonatal compartment syndrome with compound fetal presentation. It also demonstrates the need for swift, surgical intervention and multidisciplinary follow up to ensure normal recovery and development of the affected limb.

## Conclusion

Neonatal compartment syndrome is a rare, emergent complication in the newborn period that requires swift recognition, diagnosis, and surgical intervention to preserve muscle and nerve function of the affected extremity. Compound fetal presentation should be considered a cause and risk factor for the development of neonatal compartment syndrome, and clinicians should closely monitor for signs of it in children whose delivery was complicated by a compound fetal presentation. Children affected by neonatal compartment syndrome should undergo close, multidisciplinary follow up to ensure normal bone and limb development.

## Data Availability

Data used and/or analyzed during the current study are available from the corresponding author on reasonable request.

## Abbreviations

HSV-2: Herpes Simplex Virus 2
TSH: Thyroid Stimulating Hormone
IU: International unit
L: Liter
